# The beta Burr type X distribution properties with application

**DOI:** 10.1186/s40064-016-2271-9

**Published:** 2016-05-20

**Authors:** Faton Merovci, Mundher Abdullah Khaleel, Noor Akma Ibrahim, Mahendran Shitan

**Affiliations:** Department of Mathematics, Faculty of Natural Science and Mathematics, University of Mitrovica “Isa Boletini”, Str. Industrial Park, 40000 Mitrovica, Republic of Kosovo; Department of Mathematics, Faculty of Science, University Putra Malaysia, 43400 Serdang, Salangor Malaysia; Laboratory of Computational Statistics and Operations Research, Institute for Mathematical Research, University Putra Malaysia, Serdang, Malaysia

**Keywords:** Quantile function, Moment, Order statistics, Estimation

## Abstract

We develop a new continuous distribution called the beta-Burr type X distribution that extends the Burr type X distribution. The properties provide a comprehensive mathematical treatment of this distribution. Further more, various structural properties of the new distribution are derived, that includes moment generating function and the *r*th moment thus generalizing some results in the literature. We also obtain expressions for the density, moment generating function and *r*th moment of the order statistics. We consider the maximum likelihood estimation to estimate the parameters. Additionally, the asymptotic confidence intervals for the parameters are derived from the Fisher information matrix. Finally, simulation study is carried at under varying sample size to assess the performance of this model. Illustration the real dataset indicates that this new distribution can serve as a good alternative model to model positive real data in many areas.

## Background

In recent years, Burr type X (BX) distribution was introduced by Burr (1942) and it has received much attention in the literatures. The BX distribution has played an important role in reliability study, modeling the life time of random phenomena, health, agriculture and biology. Consider the two parameter Burr type X with cumulative distribution function (CDF),

1 $$F(x ,\lambda ,\theta )=\left[ 1-e^{-(\lambda x)^{2}}\right] ^{\theta }= F_{\lambda ,\theta }(x),\quad x,\theta ,\lambda >0$$

where $\theta ,\lambda$ are the shape and scale parameters, respectively. Then the probability density function (PDF) is

2 $$f(x,\theta ,\lambda )=2\theta \lambda ^{2}\,x \,e^{-(\lambda x)^{2}}\left[ 1-e^{-(\lambda x)}\right] ^{\theta -1}.$$

The *k*th moment for the BX distribution is defined as in Surles and Padgett (2005)

$$\mu ^{(k)}=\frac{\theta }{\lambda ^{k}} \Gamma \left( \frac{m}{2}+1\right) \sum ^{\theta - 1}_{j=0} {\theta -1 \atopwithdelims ()j}\frac{(-1)^{j}}{\left( j+1\right) ^{\frac{k}{2}+1}}.$$

The two parameter BX has several types of distribution like Rayleigh (R) when $(\theta = 1)$ and Burr type X distribution with one parameter (BX1) when $(\lambda =1)$. BX1 has been studied by some authors, for example: Ahmad Sartawi and Abu-Salih (1991),  Jaheen (1995),  Jaheen (1996),  Ahmad et al. (1997),  Raqab (1998) and Surles and Padgett (1998). Surles and Padgett (2001) proposed and observed that Eq. (1) could be used quite effectively in modeling strength data as well as modeling general life time data. Raqab and Kundu (2006) studied the relationship of Burr type X with Weibull, Gamma, Generalized Exponential and Exponentiated Weibull distributions.  Lio et al. (2014) studied the control charts for monitoring Burr X, and in the same year (Smith et al. 2015) studied the higher order inference for stress–strength reliability with independent Burr X.

In this article we extend the Burr type X distribution with two parameters introduced by Surles and Padgett (2001) by proposing the beta Burrtype X (BBX) distribution which contains some special sub models and it seems to be more flexible as an alternative model to use in a variety of life time problems.

The extension of this Burr type X distribution with two parameters is through the beta-G generator defined by  Eugene et al. (2002). We investigate and explore the properties of this new distribution. Eugene et al. (2002) proposed a new technique for building a new distribution from G(x). It is known as the beta generalized class of distribution and it has two shape parameters in the generator. If G is the cumulative function of any random variable, the beta generalized distribution is define by

3 $$G(x,\alpha ,\beta )=\frac{1}{B(\alpha ,\beta )} \int ^{F(x)}_{0}t^{\alpha -1}(1-t)^{\beta -1}dt \quad 0<\alpha , \beta < \infty,$$

where $\alpha$, $\beta$ are the extra shape parameters for the G distribution.

The beta function is

$$g(x,\alpha ,\beta )=\frac{1}{B(\alpha ,\beta )}x^{\alpha -1}(1-x)^{\beta -1} ,$$

where   $$0< x <1,\; \alpha>0,\; \beta >0$$ and

$$B(\alpha ,\beta )=\frac{\Gamma (\alpha ) \Gamma (\beta )}{\Gamma (\alpha +\beta )}.$$

The CDF for beta distribution is

$$G(x,\alpha ,\beta )=\frac{1}{B(\alpha ,\beta )} \int ^{x}_{0}t^{\alpha -1}(1-t)^{\beta -1}dt .$$

Another function for beta distribution is the Incomplete beta function and is defined as:

$$\begin{aligned} G(x,\alpha ,\beta )=I_{x}(\alpha ,\beta ) =\frac{B(x,\alpha ,\beta )}{B(\alpha ,\beta )} , \end{aligned}$$

where by

$$B(x,\alpha ,\beta )=\int ^{x}_{0}t^{\alpha -1}(1-t)^{\beta -1}dt .$$

Paranaíba et al. (2011) have introduced beta Burr type XII (BBXII) distribution which has five parameters (4 shape and one scale), which is different model to BBX. The BBXII has sub-models such is beta Weibull, beta Log-Logistic, beta Pareto type II, and exponentiated Burr type XII it is different from BBX as we see later.

This kind of class has received considerable attention in recent years. After the work by Eugene et al. (2002) many authors follow the same idea by taking a different G(x) such as  Nadarajah and Gupta (2004), Nadarajah and Kotz (2004, 2006),  Akinsete et al. (2008), Silva et al. (2010),  Pescim et al. (2010), Cordeiro et al. (2011, 2013),  Lemonte (2014), Domma and Condino (2013), Merovci and Sharma (2014), and  Jafari et al. (2014), among others. The rest of this paper is organized as follows: in “Beta Burr X” section, we introduce the PDF and CDF of beta Burr type X, the plot of the PDF and hazard function followed by finding the limit of Hazard function. In “Some properties of the BBX distribution” section, we discuss some important properties of the BBX. The estimation parameters by using maximum likelihood estimation (MLEs) of the unknown parameters are derived in “Parameter estimation” section. We have provided the simulation study in “Simulation study” section. The application of the model on real data set are provided in “Application” section. Finally, “Conclusion” section ends with some conclusions.

## Beta Burr X

In this section, we introduce the BBX and discuss its important properties. Suppose that *F*(*x*) is the cumulative distribution function of a random variable X. The CDF for a generalized class of distribution for the random variable of X, according to  Eugene et al. (2002), can be generated by applying the inverse CDF for a beta distribution.

### PDF, CDF, hazard function, plots, and limit

For any continuous baseline, the cumulative distribution function for the beta-G distribution G(x) is given as:

$$G(x,\alpha ,\beta )= \frac{1}{B(\alpha ,\beta )}\int ^{F(x)}_{0}t^{\alpha -1}(1-t)^{\beta -1}dt \quad 0<\alpha , \beta < \infty,$$

where $\alpha$ and $\beta$ are additional shape parameters.

The probability density function is given by $g(x)=G^{'}(x)$ meaning that

4 $$g(x,\alpha ,\beta )= \frac{1}{B(\alpha ,\beta )}\Big (F(x)\Big )^{\alpha -1} \Big (1-F(x)\Big )^{\beta -1}f(x).$$

The probability density function $f(x)=F^{'}(x)$ has been studied by many authors assuming various type of CDF of *F*(*x*). Now let suppose the *F*(*x*) is the CDF of Burr X distribution as given in Eq. (1). The *g*(*x*) for the new beta Burr type X distribution from (1) and (4) is

5 $$\begin{aligned} g(x,\alpha ,\beta ,\lambda , \theta ) &=\frac{2\theta \lambda ^{2} x}{B(\alpha ,\beta )}\left\{ \left[ 1-e^{-(\lambda x)^{2}} \right] ^{\theta }\right\} ^{\alpha -1}\left\{ 1-\left[ 1-e^{-(\lambda x)^{2}} \right] ^{\theta }\right\} ^{\beta -1}\nonumber \\&\quad *e^{-(\lambda x)^{2}}\left[ 1-e^{-(\lambda x)^{2}}\right] ^{\theta -1}. \end{aligned}$$

where $x>0,\alpha>0, \beta>0, \lambda ,\theta > 0$ that can be reduced to

6 $$g(x,\alpha ,\beta ,\lambda , \theta )=\frac{2\theta \lambda ^{2} x e^{-(\lambda x)^{2}}}{B(\alpha ,\beta )}\left[ 1-e^{-(\lambda x)^{2}}\right] ^{ \theta \alpha -1} \left\{ 1-\left[ 1-e^{-(\lambda x)^{2}}\right] ^{\theta }\right\} ^{\beta -1}.$$

If X is a random variable with PDF (6) ,then $X\sim$ BBX$(\alpha ,\beta ,\lambda ,\theta )$.

The CDF for BBX is

7 $$\begin{aligned} G(x) &= I_{F(x)}(\alpha ,\beta )=\frac{1}{B(\alpha ,\beta )} \int ^{F(x)}_{0}w^{\alpha -1}\Big (1-w\Big )^{\beta -1}dw.\nonumber \\ G(x) &= I_{\left[ 1-e^{-(\lambda x)^{2}}\right] ^{\theta }}(\alpha ,\beta )=\frac{1}{B(\alpha ,\beta )} \int ^{\left[ 1-e^{-(\lambda x)^{2}}\right] ^{\theta }}_{0}w^{\alpha -1}\Big (1-w\Big )^{\beta -1}dw. \end{aligned}$$

The hazard rate function is defined as the ratio of the density function to its survival function, so the hazard rate function of the two-parameter BBX distribution is given by

$$h_{F}(x,\alpha ,\beta ,\lambda ,\theta )=\frac{g(x)}{1-G(x)}.$$

With $$h_{F}(x)>0$$ and $$\int ^{\infty }_{0}h_{F}(x)dx=\infty$$ ,

8 $$h_{F}(x)=\frac{2\theta \lambda ^{2} x e^{-(\lambda x)^{2}} \left[ 1-e^{-(\lambda x)^{2}}\right] ^{\theta \alpha -1}}{B(\alpha ,\beta )\left\{ 1-I_{\left[ 1-e^{-(\lambda x)^{2}}\right] ^{\theta }}(\alpha ,\beta )\right\} } \left\{ 1-\left[ 1-e^{-(\lambda x)^{2}}\right] ^{\theta }\right\} ^{\beta -1} \quad x>0$$

**Fig. 1:**
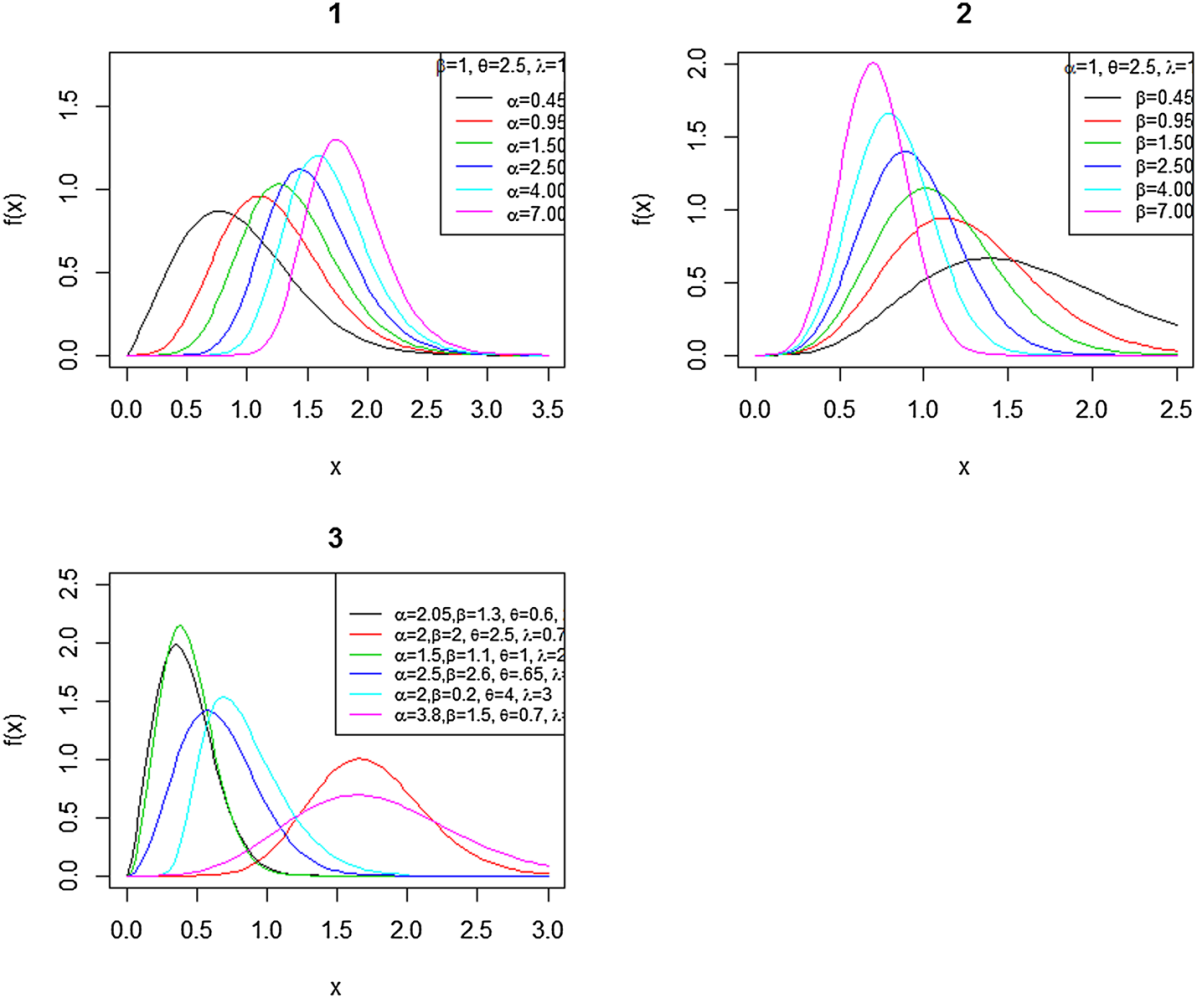
Plot of the BBX density function for some parameter values. (*1*) For different values of $\theta$ with $\alpha = 1$, $\beta =2.5,$ and $\lambda = 1$. (*2*) For different values of $\beta$ with $\alpha =1$, $\theta =2.5$, and $\lambda =1$. (*3*) For different values of $\alpha ,\beta ,\lambda$ and $\theta$

**Fig. 2:**
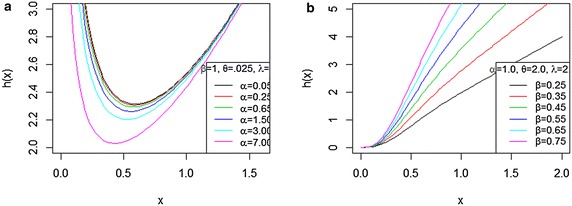
Plot of the BBX hazard function for some parameter values. **a** For different values of $\alpha$ with $\beta =1, \theta =0.025$, and $\lambda =1$. **b** For different values of $\beta$ with $\alpha =1,\theta =2$, and $\lambda =2$

Figures 1 and 2 illustrate some of possible shapes of the density and hazard functions for selected parameter values. The density and hazard functions can take many forms depending on the selected different values of parameter. The hazard function can be an increased form or bathtub shape depending on the values of parameter. The BBX distribution as we can see is more flexible than Burr type X distribution with two parameters. We can see that by the additional parameters of shape $(\alpha ,\beta )$, it allows for higher degrees of flexibility. The new model has been very useful in many partial situation like modeling positive real data set.

The limit of beta-Burr type X density function when $x\rightarrow 0$ is 0, and $x\rightarrow \infty$ is 0. We can show this by taking the limit of BBX density in Eq. (6) as follows.

$$\lim _{x \rightarrow 0} g(x) =\lim _{x \rightarrow 0} \frac{2\theta \lambda ^{2} x e^{-(\lambda x)^{2}}}{B(\alpha ,\beta )} \lim _{x \rightarrow 0}\left[ 1-e^{-(\lambda x)^{2}}\right] ^{ \theta \alpha }\lim _{x \rightarrow 0} \left\{ 1-\left[ 1-e^{-(\lambda x)^{2}}\right] ^{\theta }\right\} ^{\beta -1}=0,$$

because

$$\lim _{x \rightarrow 0} \frac{2\theta \lambda ^{2} x e^{-(\lambda x)^{2}}}{B(\alpha ,\beta )}=0 .$$

Likewise, as $x \rightarrow \infty$, we immediately can see that by replacing the limit $x \rightarrow 0$ with $x \rightarrow \infty$, the above limit expression becomes zero.

$$\lim _{x \rightarrow \infty } \frac{2\theta \lambda ^{2} x e^{-(\lambda x)^{2}}}{B(\alpha ,\beta )}=0.$$

## Some properties of the BBX distribution

We use the appropriate transformation to ease the steps of attaining the properties of BBX distribution starting from the equation of the CDF (7). First, if $\left| w\right| <1$ and $\beta >0$ is real non-integer, we have the series representation

9 $$(1-w)^{\beta -1}=\sum ^{\infty }_{j=0}\frac{(-1)^{j} \Gamma (\beta )}{\Gamma (\beta -j) j!}w^{j}.$$

We provide two simple expansions of CDF for BBX depending on the parameters ($\beta$ or $\alpha$) as real integer or non integer. We obtain expansions for G(x) in terms of an infinite of finite weighted sums of CDF of BX distributions. So, by using the expansion of (9) and $\beta >0$ real non-integer, we can write G(x) for BBX as

10 $$\begin{aligned} G(x)&=\frac{1}{B(\alpha ,\beta )}\sum ^{\infty }_{j=0} \frac{(-1)^{j}\Gamma (\beta )}{\Gamma (\beta -j) j!} \int ^{\left[ 1-e^{-(\lambda x)^{2}}\right] ^{\theta }}_{0}w^{\alpha +j-1}dw\nonumber \\&=\frac{1}{B(\alpha ,\beta )}\sum ^{\infty }_{j=0} \frac{(-1)^{j}\Gamma (\beta )\left\{ \left[ 1-e^{-(\lambda x)^{2}}\right] ^{\theta }\right\} ^{\alpha +j}}{\Gamma (\beta -j) j!(\alpha +j)} \end{aligned}$$

11 $$G(x)=\frac{\Gamma (\beta )}{B(\alpha ,\beta )} \sum ^{\infty }_{j=0}\frac{(-1)^{j}F_{\lambda ,\theta (\alpha +j)}(x)}{\Gamma (\beta -j) j!(\alpha +j)}.$$

For positive non-integer $\beta$ the expansion of (11) reveals the property that the CDF of the BBX distribution can be expressed as an infinite weighted sum of CDFs of BX distributions,

$$G(x)=\sum ^{\infty }_{j=0}w_{k}F_{\lambda ,\theta (\alpha +j)}(x),$$

where

$$w_{k}=\frac{\Gamma (\beta )(-1)^{j} }{B(\alpha ,\beta )\Gamma (\beta -j) j!(\alpha +j)}.$$

By using the binomial expansion in (3), if $\beta >0$ is an integer, the CDF for BBX can be written as

12 $$G(x)=\frac{1}{B(\alpha ,\beta )}\sum ^{\beta -1}_{j=0} {\beta -1 \atopwithdelims ()j}\frac{(-1)^{j}}{\alpha +j}\left[ 1-e^{-(\lambda x)^{2}}\right] ^{\theta (\alpha +j)}.$$

The benefit of the Eq. (12) is that we can plot the graph of G(x) with different parameter. Equations (10) and (12) are new formula of the CDF of BBX. When both $\beta$ and $\alpha = n - \beta + 1$ are integers,from the Wolfram Functions Site (http://functions.wolfram.com/GammaBetaErf/BetaRegularized/03/01/) it says that for integer $\alpha$

$$I_{y}(\alpha ,\beta )=1-\frac{(1-y)^{\beta }}{\Gamma (\beta )} \sum ^{\alpha -1}_{j=0}\frac{\Gamma (\beta +j)}{j!}y^{j},$$

and for the integer $\beta$

$$I_{y}(\alpha ,\beta )=\frac{y^{\alpha }}{\Gamma (\alpha )} \sum ^{\beta -1}_{j=0}\frac{\Gamma (\alpha +j)}{j!}(1-y)^{j} .$$

Therefore if $\alpha$ is an integer

13 $$G(x)=1-\frac{\left\{ 1-\left( 1-e^{-(\lambda x)^{2}}\right) ^{\theta }\right\} ^{\beta }}{\Gamma (\beta )} \sum ^{\alpha -1}_{j=0}\frac{\Gamma (\beta +j)}{j!}\left[ 1-e^{-(\lambda x)^{2}}\right] ^{\theta j},$$

and for $\beta > 0$ integer we have an alternative form for (11) given by

14 $$G(x)=\frac{\left\{ 1-\left[ 1-e^{-(\lambda x)^{2}}\right] ^{\theta }\right\} ^{\alpha } }{\Gamma (\alpha )}\sum ^{\beta -1}_{j=0} \frac{\Gamma (\alpha +j)}{j!}\left\{ 1-\left[ 1-e^{-(\lambda x)^{2}}\right] ^{\theta }\right\} ^{j}.$$

The following results help in the generation of observations from the BBX distribution. If *V* is a random variable following beta distribution with parameters $\alpha$ and $\beta$, then

$$X=F^{-1}(V)=\frac{\left[ -log(1-V^{\frac{1}{\theta }}) \right] ^{\frac{1}{2}}}{\lambda },$$

follows BBX distribution with parameters $\alpha ,\beta ,\lambda$ and $\theta$.

### Quantile function, skewness and kurtosis

The quantile function QF is denoted by $Q(p)=F^{-1}(p)$, so, we can compute the quantile function by inversing (7)

$$x=Q(p)=F^{-1}(p)=\frac{1}{\lambda } \left( -log\left\{ 1-\left[ I^{-1}_{p}(\alpha ,\beta )\right] ^ \frac{1}{\theta }\right\} \right) ^\frac{1}{2},$$

where $I^{-1}_{p}(\alpha ,\beta )$ is the inverse of incomplete beta function and from Wolfarm website (http://functions.wolfram.com/06.23.06.0004.01),

$$\begin{aligned} I^{-1}_{p}(\alpha ,\beta )&=w+\frac{(\beta -1)}{(\alpha +1)}w^{2}+\frac{(\beta -1)(\alpha ^{2}+3\alpha \beta -\alpha +5\beta -4)}{2(\alpha +1)^{2}(\alpha +1)}w^{3}\\&\quad +\,\frac{(\beta -1)[\alpha ^{4}+(6\beta -1)\alpha ^{3}+(\beta +2)(8\beta -5)\alpha ^{2}]}{3(\alpha +1)^{3}(\alpha +2)(\alpha +1)}w^{4}\\&\quad +\,\frac{(\beta -1)[(33\beta ^{2}-30\beta +4)\alpha +\beta (31\alpha -47)+18]}{3(\alpha +1)^{3}(\alpha +2)(\alpha +1)}w^{4} +O\left(P^{\frac{5}{\alpha }}\right), \end{aligned}$$

where $w=[\alpha p B(\alpha ,\beta )]^{1/\alpha }$ for $\alpha >0$ and $0<p<1$. From the quantile measure we can find the skewness and kurtosis. Classical kurtosis is known to have shortcomings. Due to this, we used the Bowley Skewness (Kenney and Keeping 1954) based on quartiles and is one of the earliest skewness measure, defined by

$$S_{K}=\frac{Q(3/4)+Q(1/4)-2Q(1/2)}{Q(3/4)-Q(1/4)}.$$

The Moors kurtosis (Moors 1988) based on octile of the BBX distribution and can be calculated by using the formula given below

$$M_{u}=\frac{Q(1/8)+Q(3/8)+Q(7/8)-Q(5/8)}{Q(3/4)-Q(1/4)}.$$

**Fig. 3:**
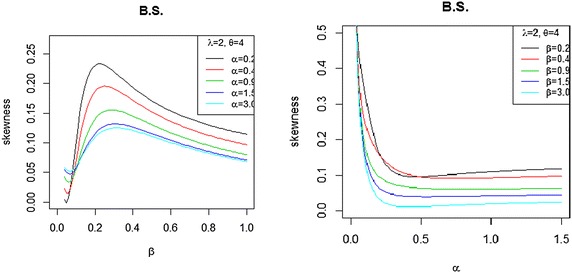
Bowley skewness of the BBX distribution as a function with different values of $\alpha$, and $\beta$ with $\lambda =2$ and $\theta =4$

**Fig. 4:**
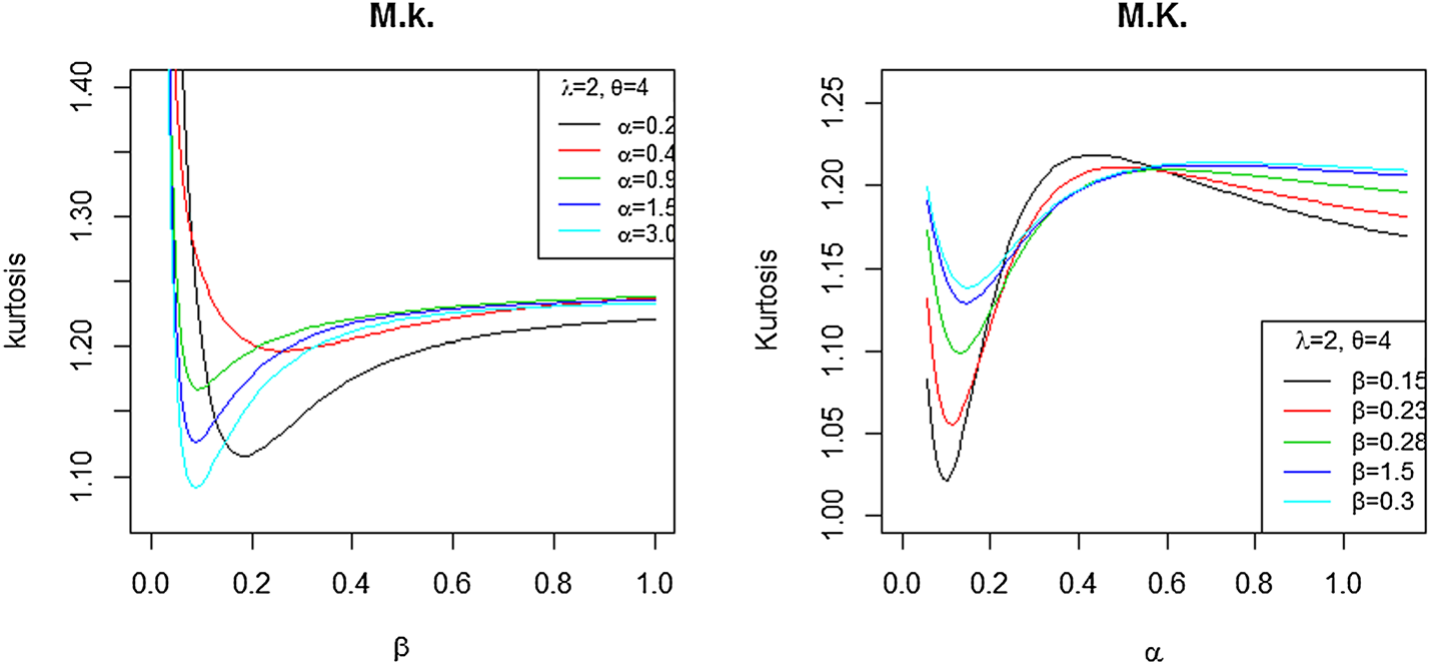
Moors kurtosis of the BBX distribution as a function with different values of $\alpha$, and $\beta$ with $\lambda =2$ and $\theta =4$

These measures are less sensitive to outliers and we can find it without moment (Figs. 3, 4).

### Sub models

The BBX has many special sub models by fixing some parameters as the follows:

- When $\alpha =\beta =1$ the BBX in Eq. (6) reduce to Burr type X with two parameters.
- When $\alpha =\beta =1,\lambda =1$ the BBX in Eq. (6) reduce to Burr type X distribution with one parameter.
- When $\alpha =\beta =1,\theta =1$ the BBX in Eq. (6) reduce to Rayleigh distribution.

### Moment and the moment generating function

In this section we find the moment and moment generating function (mgf) for BBX. Some of the most important properties of the distributions can be studied from the moments such as tendency, dispersion, skewness and kurtosis. The definition of *r*th moment of the BBX distribution is

$$E(X^{r})=\int ^{\infty }_{0}x^{r} g(x) dx ,$$

from (6) we have

$$\begin{aligned} E(X^{r})&= \int ^{\infty }_{0}x^{r}\frac{2\theta \lambda ^{2} x e^{-(\lambda x)^{2}}}{B(\alpha ,\beta )}\left[ 1-e^{-(\lambda x)^{2}}\right] ^{ \theta \alpha -1} \left\{ 1-\left[ 1-e^{-(\lambda x)^{2}}\right] ^{\theta }\right\} ^{\beta -1} dx\\&=\frac{2\theta \lambda ^{2}}{B(\alpha ,\beta )}\int ^{\infty }_{0}x^{r+1} e^{-(\lambda x)^{2}}\left[ 1-e^{-(\lambda x)^{2}}\right] ^{ \theta \alpha -1} \left\{ 1-\left[ 1-e^{-(\lambda x)^{2}}\right] ^{\theta }\right\} ^{\beta -1} dx. \end{aligned}$$

Since $0<\left[ 1-e^{-(\lambda x)^{2}}\right] ^{\theta }<1$ for $x > 0$, then by using the binomial series expansion $\left\{ 1-\left[ 1-e^{-(\lambda x)^{2}}\right] ^{\theta }\right\} ^{\beta -1}$ given by

$$\begin{aligned} (1-w)^{\beta -1}&=\sum ^{\infty }_{j=0} \frac{(-1)^{j}\Gamma (\beta )}{\Gamma (\beta -j) j!}w^{j}, \\ E(X^{r})&=\frac{2\theta \lambda ^{2}}{B(\alpha ,\beta )}\int ^{\infty }_{0}x^{r+1} e^{-(\lambda x)^{2}}\left[ 1-e^{-(\lambda x)^{2}}\right] ^{ \theta \alpha -1}\sum ^{\infty }_{j=0}\frac{(-1)^{j}\Gamma (\beta )}{\Gamma (\beta -j) j!}\left[ 1-e^{-(\lambda x)^{2}}\right] ^{\theta j} dx \\&=\frac{2\theta \lambda ^{2}}{B(\alpha ,\beta )}\int ^{\infty }_{0}x^{r+1} e^{-(\lambda x)^{2}}\sum ^{\infty }_{j=0}\frac{(-1)^{j}\Gamma (\beta )}{\Gamma (\beta -j) j!}\left[ 1-e^{-(\lambda x)^{2}}\right] ^{\theta \alpha +\theta j -1} dx \\&=\frac{2\theta \lambda ^{2}}{B(\alpha ,\beta )}\int ^{\infty }_{0}x^{r+1} e^{-(\lambda x)^{2}}\sum ^{\infty }_{j=0}\frac{(-1)^{j}\Gamma (\beta )}{\Gamma (\beta -j) j!} \left[ 1-e^{-(\lambda x)^{2}}\right] ^{\theta (\alpha + j)-1}dx \\&=\frac{2\theta \lambda ^{2}}{B(\alpha ,\beta )}\int ^{\infty }_{0}x^{r+1} e^{-(\lambda x)^{2}}\sum ^{\infty }_{j,i=0}(-1)^{j+i}\frac{\Gamma (\beta )\Gamma (\theta (\alpha + j))}{\Gamma (\beta -j)\Gamma (\theta (\alpha + j)-i) i! j!}\left[ e^{-(\lambda x)^{2}}\right] ^{i}dx \\&=\frac{2\theta \lambda ^{2}}{B(\alpha ,\beta )}\sum ^{\infty }_{j,i=0}(-1)^{j+i}\sum ^{\infty }_{j,i=0}(-1)^{j+i}\frac{\Gamma (\beta )\Gamma (\theta (\alpha + j))}{\Gamma (\beta -j)\Gamma (\theta (\alpha + j)-i) i! j!}\int ^{\infty }_{0}x^{r+1}\left[ e^{-(\lambda x)^{2}}\right] ^{i+1}dx \\&=\frac{2\theta \lambda ^{2}}{B(\alpha ,\beta )}\sum ^{\infty }_{j,i=0}(-1)^{j+i}\frac{\Gamma (\beta )\Gamma (\theta (\alpha + j))}{\Gamma (\beta -j)\Gamma (\theta (\alpha + j)-i) i! j!}\int ^{\infty }_{0} x^{r+1} \left[ e^{-(i+1)(\lambda x)^{2}}\right] dx \\&=\frac{2\theta \lambda ^{2}}{B(\alpha ,\beta )}\sum ^{\infty }_{j,i=0}(-1)^{j+i}\frac{\Gamma (\beta )\Gamma (\theta (\alpha + j))}{\Gamma (\beta -j)\Gamma (\theta (\alpha + j)-i) i! j!}\int ^{\infty }_{0} x^{r+1} e^{-(i+1)(\lambda x)^{2}}dx. \end{aligned}$$

We want to find

$$\int ^{\infty }_{0} x^{r+1} e^{-(i+1)(\lambda x)^{2}}dx.$$

Let

$$y=(i+1)(\lambda x)^{2} \Rightarrow x=\frac{y^{1/2}}{\lambda (i+1)^{1/2}} \Rightarrow dx=\frac{y^-{1/2}}{2\lambda (i+1)^{1/2}}dy$$

so

$$\begin{aligned} \int ^{\infty }_{0} x^{r+1} e^{-(i+1)(\lambda x)^{2}}dx&=\int ^{\infty }_{0}\frac{y^{\frac{r}{2}+\frac{1}{2}}}{\lambda ^{r+1}(i+1)^{\frac{r}{2}+\frac{1}{2}}} e^{-y}\frac{y^-{1/2}}{2\lambda (i+1)^{1/2}}dy\\&=\frac{1}{2\lambda ^{r+2}(i+1)^{\frac{r}{2}+1}}\int ^{\infty }_{0}y^{\frac{r}{2}}e^{-y}dy\\&=\frac{\Gamma (\frac{r}{2}+1)}{2\lambda ^{r+2}(i+1)^{\frac{r}{2}+1}}, \end{aligned}$$

so the $E(X^{r})$ is

15 $$\mu ^{(r)}=E(X^{r})=\frac{\theta \Gamma (\frac{r}{2}+1)}{\lambda ^{r} B(\alpha ,\beta )}\sum ^{\infty }_{j,i=0}\frac{(-1)^{j+i}\Gamma (\beta )\Gamma (\theta (\alpha + j))}{\Gamma (\beta -j)\Gamma (\theta (\alpha + j)-i) i! j!(i+1)^{\frac{r}{2}+1}}.$$

This is the general formula for the *r*th moment of the BBX distribution.

If $\beta >0$ is an integer, by applying the binomial expansion, we get

16 $$E(X^{r})=\frac{\theta \Gamma (\frac{r}{2}+1)}{\lambda ^{r} B(\alpha ,\beta )}\sum ^{\beta -1}_{j=0}{\beta -1 \atopwithdelims ()j} \sum ^{\infty }_{i=0}\frac{(-1)^{j+i}\Gamma (\theta (\alpha + j))}{\Gamma (\theta (\alpha + j)-i) i! (i+1)^{\frac{r}{2}+1}}.$$

Also if $\alpha >0$ is an integer then by applying binomial expansion, Eq. (16) is

17 $$E(X^{r})=\frac{\theta \Gamma (\frac{r}{2}+1)}{\lambda ^{r} B(\alpha ,\beta )}\sum ^{\beta -1}_{j=0}{\beta -1 \atopwithdelims ()j} \sum ^{(\theta (\alpha + j)-1}_{i=0}{(\theta (\alpha + j)-1 \atopwithdelims ()i}\frac{(-1)^{j+i}}{(i+1)^{\frac{r}{2}+1}}.$$

If $\beta =\alpha =1$ from Eq. (15)

18 $$E(X^{r})=\frac{\theta \Gamma (\frac{r}{2}+1)}{\lambda ^{r}} \sum ^{\theta - 1}_{i=0} {\theta -1 \atopwithdelims ()i}\frac{(-1)^{i}}{(i+1)^{\frac{r}{2}+1}}.$$

This is the *r*th moment for Burr type X distribution which is given by Surles and Padgett (2005) with parameter $\alpha$, and $\lambda$

### Order statistics

Let $X_{1}, X_{2},\ldots ,X_{n}$ be a random sample of size *n* from $X\sim$ BBX$(\alpha ,\beta ,\lambda ,\theta )$. We want to find the *i*th order stiatistics for the density function $f_{i:n}(x)$ for $i=1,2,\ldots , n$. It is well known that

$$f_{i:n}(x)=\frac{f(x)}{B(i,n-i+1)} \Big (F(x)\Big )^{i-1}\Big (1-F(x)\Big )^{n-i}.$$

By using binomial expansion we get

$$f_{i:n}(x)=\sum ^{n-i}_{l=0}\frac{(-1)^{l} {n-i \atopwithdelims ()l}f(x)}{B(i,n-i+1)} \Big (F(x)\Big )^{i+l-1}.$$

From the Eqs. (6) and (10) respectively, we have

$$\begin{aligned} f_{i:n}(x)&=\sum ^{n-i}_{l=0}\frac{(-1)^{l} {n-i \atopwithdelims ()l} (\Gamma (\beta ))^{i+l-1}2\theta \lambda ^{2} x e^{-(\lambda x)^{2}}}{B(i,n-i+1)B(\alpha ,\beta )} \left[ 1-e^{-(\lambda x)^{2}}\right] ^{ \theta \alpha (i+l)-1} \left\{ 1-\left[ 1-e^{-(\lambda x)^{2}}\right] ^{\theta }\right\} ^{\beta -1}\nonumber \\&\quad *\left\{ \sum ^{\infty }_{j=0}\frac{(-1)^{j}\left[ 1-e^{-(\lambda x)^{ 2}}\right] ^{\theta j}}{\Gamma (\beta -j) j! (\alpha +j)}\right\} ^{i+l-1}. \end{aligned}$$

By using the equation

19 $$\left( \sum ^{\infty }_{i=0}\alpha _{i}\right) ^{k} = \sum ^{\infty }_{m_{1}=0}\cdots \sum ^{\infty }_{m_{k}=0} \alpha _{m_{1}}\cdots \alpha _{m_{k}},$$

for $k>0$, we can write the PDF for order statistics as

20 $$\begin{aligned} f_{i:n}(x)&=\sum ^{n-i}_{l=0}\sum ^{\infty }_{m_{1}=0}\cdots \sum ^{\infty }_{m_{i+l-1}=0}\gamma _{i,l} f_{i,l}(x), \nonumber \\ f_{i,l}(x)&=\frac{2\theta \lambda ^{2} x e^{-(\lambda x)^{2}}\left\{ \left[ 1-e^{-(\lambda x)^{2}}\right] ^{\theta }\right\} ^{\alpha (i+l)+\theta \sum ^{i+l-1}_{j=0}m_{j}-1}}{B(\alpha (i+l)+\sum ^{i+l-1}_{j=0}m_{j},\beta )}\nonumber \\&\quad *\left\{ 1-\left[ 1-e^{-(\lambda x)^{2}}\right] ^{\theta }\right\} ^{\beta -1}, \end{aligned}$$

and

$$\gamma _{i,l}=\frac{(-1)^{l+\sum ^{i+l-1}_{j=1}m_{j}} {n-i \atopwithdelims ()l}(\Gamma (\beta ))^{i+l-1}B(\alpha (i+l)+\sum ^{i+l-1}_{j=0}m_{j},\beta )}{B(\alpha ,\beta )^{i+l-1}B(i,n-i+1)\prod ^{i+l-1}_{j=1}\Gamma (\beta -m_{j}) m_{j} (\alpha +m_{j})},$$

$f_{i,l}(x)$ is the PDF for BBX with new parameters $(\alpha (i+l)+\sum ^{i+l-1}_{j=0}m_{j},\beta , \lambda , \theta )$. Equation (20) is very important as we can find several mathematical properties for BBX order statistics like (mgf , factorial moment, ordinary moment and inverse). Thus from $f_{i:n}(x)$ we can find the $S_{th}$ moment of $X_{i:n}$.

For $\beta >0$, real non-integer then

$$E(X^{s}_{i:n})=\sum ^{n-i}_{l=0}\sum ^{\infty }_{m_{1}=0}\cdots \sum ^{\infty }_{m_{i+l-1}=0}\gamma _{i,l} E(X^{s}_{i:l}),$$

and for $\beta >0$ is an integer,

$$E(X^{s}_{i:n})=\sum ^{n-i}_{l=0}\sum ^{\beta - 1}_{m_{1}=0}\cdots \sum ^{\beta -1}_{m_{i+l-1}=0}\gamma _{i,l} E(X^{s}_{i:l}),$$

where the moments $E(X^{s}_{i:l})$ come from the general expansions of (15) and (16) for the moments of the BBX distribution with parameters $(\alpha (i+l)+\sum ^{i+l-1}_{j=0}m_{j},\beta , \lambda , \theta )$.

## Parameter estimation

The most widely used method for the estimation of parameters of distribution is the maximum likelihood estimation method (MLE) and the moment method. We employ the maximum likelihood estimation method MLE to estimate the unknown parameter of BBX distribution.

Let $X_{1},X_{2}, \ldots ,X_{n}$ be a random sample of size *n* from BBX $\left( \alpha ,\beta ,\lambda ,\theta \right)$ distribution. The likelihood function is given by

21 $$\begin{aligned} L(\alpha ,\beta ,\lambda ,\theta&=\frac{2^{n}\theta ^{n}\lambda ^{2 n}x^{n}e^{-\sum ^{n}_{i=1}(\lambda x _{i})^{2}}\left[ \Gamma (\alpha +\beta )\right] ^{n}}{\left[ \Gamma (\alpha )\right] ^{n} \left( \Gamma (\beta )\right) ^{n}} \prod ^{n}_{i=1} \left[ 1-e^{-\left( \lambda x_{i}\right] ^{2}}\right] ^{(\alpha \theta -1)} \nonumber \\&\quad *\prod ^{n}_{i=1}\left\{ 1-\left[ 1-e^{-(\lambda x _{i})^{2}}\right] ^{\theta }\right\} ^{\beta -1}\,\,. \end{aligned}$$

The log-likelihood function for the vector of parameters $\Theta =(\alpha ,\beta ,\lambda ,\theta )^{T}$ is expressed as

22 $$\begin{aligned} l=l(\Theta )&= n[\log 2 +\log \theta + 2 \log \lambda + \log x + \log \Gamma (\alpha +\beta )-\log \Gamma (\alpha )\nonumber \\&\quad - \,\log \Gamma (\beta )] -\sum ^{n}_{i=1}(\lambda x _{i})^{2}+(\alpha \theta -1)\sum ^{n}_{i=1}\log \left[ 1-e^{-(\lambda x _{i})^{2}}\right] \nonumber \\&\quad +\,(\beta -1)\sum ^{n}_{i=1}\log \left[ 1-\left( 1-e^{-(\lambda x _{i})^{2}}\right) ^{\theta }\right] . \end{aligned}$$

By taking partial derivatives of log-likelihood in (22) with respect to $\alpha ,\beta ,\lambda ,$ and $\theta$ and equating the derivatives to zero we get.

$$\begin{aligned} \frac{\partial l}{\partial \alpha }&= n\Psi (\alpha +\beta )-n\Psi (\alpha )+\theta \sum ^{n}_{i=1}\log \Big [1-e^{-(\lambda x_{i})^{2}}\Big ]=0. \\ \frac{\partial l}{\partial \beta }&= n\Psi (\alpha +\beta )-n\Psi (\beta )+\sum ^{n}_{i=1}\log \left\{ 1-\left[ 1-e^{-(\lambda x_{i})^{2}}\right] ^{\theta }\right\} =0. \\ \frac{\partial l}{\partial \lambda }&=\frac{2n}{\lambda }-2\lambda \sum ^{n}_{i=1}(x_{i})^{2}+(\alpha \theta -1)\sum ^{n}_{i=1}\frac{2\lambda (x_{i})^{2} e^{-(\lambda x_{i})^{2}}}{1-e^{-(\lambda x_{i})^{2}}} \\&\quad -(\beta -1)\sum ^{n}_{i=1}\frac{2\theta \lambda (x_{i})^{2}\left[ 1-e^{-(\lambda x_{i})^{2}}\right] ^{(\theta -1)}e^{-(\lambda x_{i})^{2}}}{\left\{ 1-\left[ 1-e^{-(\lambda x_{i})^{2}}\right] ^{\theta }\right\} }=0.\\ \frac{\partial l}{\partial \theta }&=\frac{n}{\theta }+\theta \sum ^{n}_{i=1}\log \left[ 1-e^{-(\lambda x_{i})^{2}}\right] \\&\quad -(\beta -1)\sum ^{n}_{i=1}\frac{\left[ 1-e^{-(\lambda x_{i})^{2}}\right] ^{\theta }\log \left[ 1-e^{-(\lambda x_{i})^{2}}\right] }{\left\{ 1-\left[ 1-e^{-(\lambda x_{i})^{2}}\right] ^{\theta }\right\} }=0. \end{aligned}$$

The expected value of the unit score vector vanishes leading to the following equations

23 $$E\left\{ \sum ^{n}_{i=1}\log \left[ 1-e^{-(\lambda x_{i})^{2}}\right] \right\} =\frac{n\Psi (\alpha )-n\Psi (\alpha +\beta )}{\theta }.$$

24 $$E\left\{ \sum ^{n}_{i=1}\log \left[ 1-\left( 1-e^{-(\lambda x_{i})^{2}}\right) ^{\theta }\right] \right\} =n\Psi (\beta )-n\Psi (\alpha +\beta ) .$$

25 $$E\left\{ \sum ^{n}_{i=1}\frac{\left( 1-e^{-(\lambda x_{i})^{2}}\right) ^{\theta }\log \left( 1-e^{-(\lambda x_{i})^{2}}\right) }{\left\{ 1-\left[ 1-e^{-(\lambda x_{i})^{2}}\right] ^{\theta }\right\} }\right\} =\frac{n\left( 1-\theta [\Psi (\alpha )-\Psi (\alpha +\beta )]\right) }{\theta (\beta -1)}.$$

It is impossible to solve Eqs. 23–25 algebraically to obtain the MLEs for $\alpha ,\beta ,\lambda ,$ and $\theta$. We can use software to obtain the MLE’s numerically like NR (Newton–Raphson), Limited-Memory quasi-Newton code for Bound-constrained optimization (L-BFGS-B), BFGS (Broyden–Fletcher–Goldfarb–Shanno), SANN (Simulated-Annealing) and, BHHH (Berndt–Hall–Hall–Hausman). In the literature, there are authors who have developed new alternative of neural network for the parameter estimates of Burr family distributions see  Abbasi et al. (2010),  Zoraghi et al. (2012)

For interval estimation and test hypotheses on the parameter, we obtain the observed information matrix $4\times 4$ where

$$\begin{aligned} J &= J(\phi ),\quad\phi =(\alpha ,\beta ,\lambda , \theta )^{T}.\\ J(\phi ) &= - \begin{pmatrix} L_{\alpha \alpha } &\quad L_{\alpha \beta } &\quad L_{\alpha \lambda } &\quad L_{\alpha \theta } \\ \cdot &\quad L_{\beta \beta } &\quad L_{\beta \lambda } &\quad L_{\beta \theta } \\ \cdot &\quad \cdot &\quad L_{\lambda \lambda } &\quad L_{\lambda \theta } \\ \cdot &\quad \cdot &\quad \cdot &\quad L_{\theta \theta } \end{pmatrix}, \end{aligned}$$

whose elements are given in “Appendix”. Under conditions that are fulfilled for parameters in the interior of the parameter space, the asymptotic distribution of $\sqrt{n}(\hat{\phi }-\phi )$ is multivariate normal $N_{4}(0,J(\phi )^{-1})$. The asymptotic $N_{4}(0,J(\hat{\phi })^{-1})$ distribution can be used to construct approximate confidence intervals and confidence regions for the parameters. Here, $J\hat{(\phi )}$ is the total observed information matrix evaluated at $\hat{\phi }$. The asymptotic $100(1-\eta )\%$ confidence intervals for $\alpha ,\beta ,\lambda$ and $\theta$ are given by $\hat{\alpha }\pm z_{\eta /2} \times \sqrt{var(\hat{\alpha })}$, $\hat{\beta }\pm z_{\eta /2} \times \sqrt{var(\hat{\beta })}$, $\hat{\lambda }\pm z_{\eta /2} \times \sqrt{var(\hat{\lambda })}$ and $\hat{\theta }\pm z_{\eta /2} \times \sqrt{var(\hat{\theta })}$ respectively, where var(.) is the diagonal element of $N_{4}(0,J(\hat{\phi })^{-1})$ corresponding to each parameter and $z_{\eta /2}$ is the quantile $100(1-\eta )\%$ of the standard normal distribution. The likelihood ratio (LR) statistic is useful for testing goodness of fit of the BBX distribution and for comparing this distribution with some of its special sub models like Burr X one parameter, Burr X two parameter, Rayleigh, and Exponential. We can compute the maximum values of the unrestricted and restricted log-likelihoods to construct LR statistics for testing some sub-models of the BBX distribution. For example, we may use the LR statistic to check if the fit using the BBX distribution is statistically “superior” to a fit using the Burr X distribution for a given data set. In any case, hypothesis tests of the type $H_{\circ }:\phi =\phi _{\circ }$ versus $H_{1}:\phi \ne \phi _{\circ }$ can be performed using LR statistics. In this case, the LR statistic for testing $H_{\circ }$ versus $H_{1}$ is $\omega =2[L(\hat{\phi })-L(\hat{\phi _{\circ }})]$ where the $\hat{\phi }$ and $\hat{\phi _{\circ }}$ are the MLEs under $H_{1}$ and $H_{\circ }$ respectively. The statistics $\omega$ is asymptotically $(n\rightarrow \infty )$ distributed as $\chi ^{2}_{k}$ where k is the dimension of the subset $\Omega$ of interest. The LR test rejects $H_{\circ }$ if the $\omega > \zeta _{\eta }$ where $\zeta _{\eta }$ denote the upper $100\eta \%$ point of the $\chi ^{2}_{k}$ distribution.

## Simulation study

**Fig. 5:**
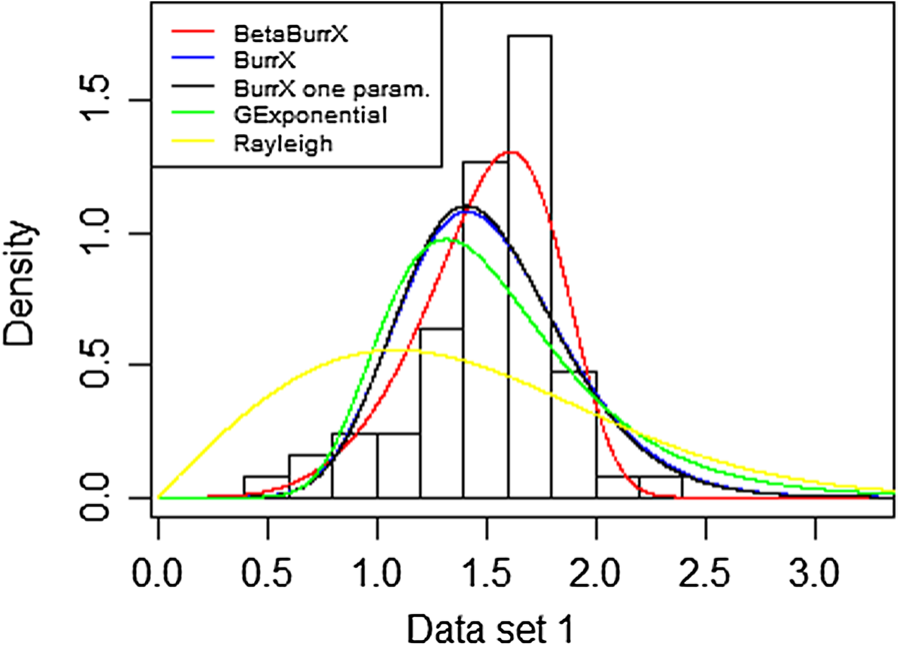
Beta Burr type X and its sub models for the strengths of 1.5 cm glass fibers

We consider Monte Carlo simulation studies to asses the performance of the MLEs of $\alpha , \beta , \lambda$ and $\theta$. We carry out using the software R simulation by generating different *n* observation from BBX distribution. The parameters are estimated by maximum likelihood method. We considered different sample size *n* = 100, 500, 1000 and 1500 and the number of repetition is 5000. The true parameters value as $\alpha = 0.5, \beta = 0.2, \lambda = 0.5$ and $\theta = 8$. Table 1 listed the bias and root mean squared error (RMSE) of the estimate parameters. We observed that, when we increase *n* the bias for $\hat{\alpha }$, $\hat{\lambda }$, $\hat{\beta }$ and $\hat{\theta }$ are very small or close to zero also the RMSE be very small (Fig. 5).

**Table 1:** Bias and root mean squared error on Monte Carlo simulation when $\alpha =0.5$, $\beta =0.2$, $\lambda =0.5$ and $\theta =8$

*n*	Parameter	Bias	RMSE
100	$\hat{\alpha }$	3.34 × 10^−2^	4.73 × 10^−4^
	$\hat{\beta }$	−7.47 × 10^−3^	1.05 × 10^−4^
	$\hat{\lambda }$	−1.93 × 10^−3^	2.73 × 10^−5^
	$\hat{\theta }$	2.09 × 10^−4^	2.95 × 10^−6^
500	$\hat{\alpha }$	1.13 × 10^−2^	1.60 × 10^−4^
	$\hat{\beta }$	−6.52 × 10^−4^	9.23 × 10^−5^
	$\hat{\lambda }$	−3.84 × 10^−3^	5.43 × 10^−5^
	$\hat{\theta }$	−8.10 × 10^−7^	1.13 × 10^−8^
1000	$\hat{\alpha }$	−1.63 × 10^−2^	2.31 × 10^−4^
	$\hat{\beta }$	−9.48 × 10^−3^	1.34 × 10^−4^
	$\hat{\lambda }$	−2.12 × 10^−4^	3.01 × 10^−6^
	$\hat{\theta }$	4.65 × 10^−7^	6.57 × 10^−9^
1500	$\hat{\alpha }$	−1.24 × 10^−8^	1.76 × 10^−10^
	$\hat{\beta }$	5.42 × 10^−8^	7.66 × 10^−10^
	$\hat{\lambda }$	6.58 × 10^−5^	9.31 × 10^−6^
	$\hat{\theta }$	−1.13 × 10^−9^	1.60 × 10^−11^

## Application

In this section, we use a real data set to illustrate that the beta-Burr X distribution is a better model than Burr type X, Burr type X one parameter, Generalized Exponential, and Rayleigh distributions. The description of the data are as follows:

This data set consists of 63 observations of the strengths of 1.5 cm glass fibers, originally obtained by workers at the UK National Physical Laboratory. Unfortunately, the units of measurement are not given in the paper. The data are: 0.55, 0.74, 0.77, 0.81, 0.84, 0.93, 1.04, 1.11, 1.13, 1.24, 1.25, 1.27, 1.28, 1.29, 1.30, 1.36, 1.39, 1.42, 1.48, 1.48, 1.49, 1.49, 1.50, 1.50, 1.51, 1.52, 1.53, 1.54, 1.55, 1.55, 1.58, 1.59, 1.60, 1.61, 1.61, 1.61, 1.61, 1.62, 1.62, 1.63, 1.64, 1.66, 1.66, 1.66, 1.67, 1.68, 1.68, 1.69, 1.70, 1.70, 1.73, 1.76, 1.76, 1.77, 1.78, 1.81, 1.82, 1.84, 1.84, 1.89, 2.00, 2.01, 2.24. These data have also been analyzed by Smith and Naylor (1987)

**Table 2:** The ML estimates, log-likelihood, AIC, CAIC and BIC for data set

Model	ML estim.	LL	AIC	CAIC	BIC
Beta Burr type X	$\hat{\alpha }= -0.7249$	16.0016	40.003	40.691	48.578
	$$\hat{\beta }= 13.3691$$				
	$$\hat{\lambda }= 0.28056$$				
	$$\hat{\theta }=7.81788$$				
Burr type X	$\hat{\lambda }= 0.9869$	23.9287	51.8575	52.0575	56.1437
	$$\hat{\theta }= 5.4860$$				
Burr type X one parameter	$\hat{\lambda }= 5.7249$	23.9584	49.9167	49.9823	56.0599
G Exponential	$\hat{\theta }= 2.6115$	31.3834	66.7669	66.9669	71.0532
	$$\hat{\lambda }= 31.3489$$				
Rayleigh	$\hat{\theta }= 0.6490$	49.7909	101.5818	101.6474	103.7249

In order to compare all the distribution models, we consider criteria like log likelihood (LL), Akaike Information Criterion (AIC), Consistent Akaike Information Criterion (CAIC) and Bayesian information criterion (BIC) for the data set. The better distribution corresponds to smaller LL, AIC, AICC and BIC values. The distribution of the data is skewed to the left (skewness = –0.95 and kurtosis = 1.01). This suggest that the BBX distribution is very good in modeling left skewed data.

The 95 % confidence interval for $\hat{\alpha }, \hat{\beta }, \hat{\lambda }$ and $\hat{\theta }$ are [−0.005175, 0.841059], [−200.0489, 358.5764], [0.180912, 0.826159] and [0.690502, 14.140148] respectively.

The LR test statistic to test the hypotheses $H_0: a=b=1$ versus $H_1: a \not =1\vee b\not =1$. For this data set $\omega = 18.671> 5.991 = \chi ^2_{2; 0.05}$, so we reject the null hypothesis.

Table 2 shows MLEs for each one of the two fitted distributions for data set and the values of LL, AIC, CAIC and BIC values. The values in the Table 2, indicate that the beta Burr X is a strong competitor to other distributions used here for fitting the data set. A density plot compares the fitted densities of the models with the empirical histogram of the observed data. The fitted density for the beta Burr X model is closer to the histogram than the fits of the other sub models.

## Conclusion

In this paper, we proposed a new distribution which generalizes the Burr type X distribution. We named is beta Burr type X and it has a special sub models. The CDF, PDF, hazard function and limit of PDF are derived. Additionally, some of the mathematical and statistical properties like quantile function, skewness, kurtosis, *r*th moment and order statistic are also provided. The model parameters are estimated by using maximum likelihood estimation and we derived the observed information matrix. Simulation study is carried at under varying sample size to assess the performance of this model. Finally, application of a real data set by using the goodness of fit is illustrated. This new distribution provides a better fit than its sub models and it is very good model for left skewed data.

## Appendix

The elements of the $4\times 4$ unit expected information matrix are given by:

$$\begin{aligned} L_{\alpha \alpha }&= n\left[ \psi ^{'}(\alpha +\beta )-\psi ^{'}(\alpha )\right] , \\ L_{\beta \beta }&=n\left[ \psi ^{'}(\alpha +\beta )-\psi ^{'}(\beta )\right] , \\ L_{\alpha \beta }&=n\left[ \psi ^{'}(\alpha +\beta )\right] ,\\ L_{\alpha \lambda }&=\theta \sum ^{n}_{i=1}\frac{2 \lambda x^{2}_{i}e^{-(\lambda x_{i})^{2}}}{1-e^{-(\lambda x_{i})^{2}}},\\ L_{\alpha \theta }&= \sum ^{n}_{i=1}\log \left[ 1-e^{-(\lambda x_{i})^{2}}\right] ,\\ L_{\beta \lambda }&=\sum ^{n}_{i=1}\frac{2\theta \lambda x_{i}e^{-(\lambda x_{i})^{2}}\left[ 1-e^{-(\lambda x_{i})^{2}}\right] ^{\theta -1}}{\left\{ 1-\left[ 1-e^{-(\lambda x_{i})^{2}}\right] ^{\theta }\right\} }, \\ L_{\beta \theta }&=-\sum ^{n}_{i=1}\frac{\left[ 1-e^{-(\lambda x_{i})^{2}}\right] ^{\theta }\log 1-\left[ 1-e^{-(\lambda x_{i})^{2}}\right] }{\left\{ 1-\left[ 1-e^{-(\lambda x_{i})^{2}}\right] ^{\theta }\right\} } ,\\ L_{\lambda \lambda }&=-\frac{2n}{\lambda ^{2}}-2\sum ^{n}_{i=1}x_{i}^{2} -2(\alpha \theta -1)\sum ^{n}_{i=1}\left\{ \frac{-2\lambda ^{2}(x_{i})^{4}e^{-(\lambda x_{i})^{2}}}{1-e^{-(\lambda x_{i})^{2}}}+\frac{x^{2}_{i}e^{-(\lambda x_{i})^{2}}}{1-e^{-(\lambda x_{i})^{2}}} -\frac{2\lambda ^{2}(x_{i})^{4}\left[ e^{-(\lambda x_{i})^{2}}\right] ^{2}}{\left[ 1- e^{-(\lambda x_{i})^{2}}\right] ^{2}}\right\} \\&\quad -\,2(\beta -1)\sum ^{n}_{i=1}\left\{ \frac{2(\theta ^{2}-\theta )\lambda ^{2}(x_{i})^{4}\left[ e^{-(\lambda x_{i})^{2}}\right] ^{2}\left[ 1-e^{-(\lambda x_{i})^{2}}\right] ^{(\theta -2)}}{1-\left[ 1-e^{-(\lambda x_{i}]^{2}}\right) ^{\theta }}- \frac{2\theta \lambda ^{2}(x_{i})^{4}e^{-(\lambda x_{i})^{2}}\left[ 1-e^{-(\lambda x_{i})^{2}}\right] ^{(\theta -1)}}{1-\left[ 1-e^{-(\lambda x_{i})^{2}}\right] ^{\theta }}\right\} \\&\quad -\,2(\beta -1)\sum ^{n}_{i=1}\left\{ \frac{\theta x^{2}_{i}e^{-(\lambda x_{i})^{2}}\left[ 1-e^{-(\lambda x_{i})^{2}}\right] }{1-\left[ 1-e^{-(\lambda x_{i})^{2}}\right] ^{\theta }}+\frac{2\theta ^{2}\lambda ^{2}(x_{i})^{4}\left[ 1-e^{-(\lambda x_{i})^{2}}\right] ^{(2\theta -2)}}{\left\{ 1-\left[ 1-e^{-(\lambda x_{i})^{2}}\right] ^{\theta }\right\} ^{2}}\right\} ,\\ L_{\lambda \theta }&=\sum ^{n}_{i=1}\frac{-2\theta \lambda (x_{i})^{2}e^{-(\lambda x_{i})^{2}}}{\left[ 1-e^{-(\lambda x_{i})^{2}}\right] } - 2(\beta -1) \left\{ \frac{\lambda (x_{i})^{2}e^{-(\lambda x_{i})^{2}}\left[ 1-e^{-(\lambda x_{i})^{2}}\right] ^{(\theta -1)}}{1-\left[ 1-e^{-(\lambda x_{i})^{2}}\right] ^{\theta }}\right\} ,\\&\quad -\,2(\beta -1)\left\{ \frac{\theta \lambda (x_{i})^{2}e^{-(\lambda x_{i})^{2}}\left[ 1-e^{-(\lambda x_{i})^{2}}\right] ^{(\theta -1)}\log \left[ 1-e^{-(\lambda x_{i})^{2}}\right] }{1-\left[ 1-e^{-(\lambda x_{i})^{2}}\right] ^{\theta }}\right\} ,\\&\quad -\,2(\beta -1)\left\{ \frac{\theta \lambda (x_{i})^{2}e^{-(\lambda x_{i})^{2}}\left[ 1-e^{-(\lambda x_{i})^{2}}\right] ^{(2\theta -1)}\log \left[ 1-e^{-(\lambda x_{i})^{2}}\right] }{\left\{ 1-\left[ 1-e^{-(\lambda x_{i})^{2}}\right] ^{\theta }\right\} ^{2}}\right\} ,\\ L_{\theta \theta }&=-\sum ^{n}_{i=1}(\beta -1)\left\{ \frac{\left[ 1-e^{-(\lambda x_{i})^{2}}\right] ^{\theta } \log \left[ 1-e^{-(\lambda x_{i})^{2}}\right] ^{2}}{1-\left[ 1-e^{-(\lambda x_{i})^{2}}\right] ^{\theta }}+\frac{\left[ 1-e^{-(\lambda x_{i})^{2}}\right] ^{(2 \theta )}\log \left[ 1-e^{-(\lambda x_{i})^{2}}\right] ^{2}}{\left\{ 1-\left[ 1-e^{-(\lambda x_{i})^{2}}\right] ^{\theta }\right\} ^{2}}\right\} . \end{aligned}$$
